# Utility of Simultaneous Left Atrial Strain–Volume Relationship During Passive Leg Lift to Identify Elevated Left Ventricular Filling Pressure—A Proof-of-Concept Study

**DOI:** 10.3390/jcm13247629

**Published:** 2024-12-14

**Authors:** Ashwin Venkateshvaran, Urban Wiklund, Per Lindqvist, Thomas Lindow

**Affiliations:** 1 Clinical Physiology, Department of Clinical Sciences Lund, Skåne University Hospital, Lund University, 221 84 Lund, Sweden; 2 Department of Diagnostics and Intervention, Biomedical Engineering and Radiation Physics, Umeå University, 901 87 Umeå, Sweden; urban.wiklund@umu.se; 3 Department of Diagnostics and Intervention, Clinical Physiology, Umeå University, 901 87 Umeå, Sweden; per.lindqvist@umu.se; 4 Department of Clinical Physiology, Research and Development, Region Kronoberg, Växjö Central Hospital, 352 34 Växjö, Sweden; 5 Pulmonary Medicine, Allergology, and Palliative Medicine, Department of Clinical Sciences Lund, Lund University, 221 84 Lund, Sweden

**Keywords:** heart failure, speckle-tracking echocardiography, pulmonary capillary wedge pressure

## Abstract

**Background**: The assessment of left ventricular (LV) filling pressure in heart failure (HF) poses a diagnostic challenge, as HF patients may have normal LV filling pressures at rest but often display elevated LV filling pressures during exercise. Rapid preload increase during passive leg lift (PLL) may unmask HF in such challenging scenarios. We explored the dynamic interplay between simultaneous left atrial (LA) function and volume using LA strain/volume loops during rest and PLL and compared its diagnostic performance with conventional echocardiographic surrogates to detect elevated LV filling pressure. **Methods**: We retrospectively reviewed 35 patients with clinical HF who underwent simultaneous echocardiography and right heart catheterization before and immediately after PLL. Patients with atrial fibrillation (*n* = 4) were excluded. Twenty age-matched, healthy controls were added as controls. LA reservoir strain (LASr) was analyzed using speckle-tracking echocardiography. LA strain–volume loops were generated, including the best-fit linear regression line employing simultaneous LASr and LA volume. **Results**: LA strain–volume slope was lower for HF patients when compared with controls (0.71 vs. 1.22%/mL, *p* < 0.001). During PLL, the LA strain–volume slope displayed a moderately strong negative correlation with invasive pulmonary arterial wedge pressure (PAWP) (r = −0.71, *p* < 0.001). At a 0.74%/mL cut-off, the LA strain–volume slope displayed 88% sensitivity and 86% specificity to identify elevated PAWP (AUC 0.89 [0.76–1.00]). In comparison, LASr demonstrated strong but numerically lower diagnostic performance (AUC 0.82 [0.67–0.98]), and mitral E/e’ showed poor performance (AUC 0.57 [0.32–0.82]). **Conclusions**: In this proof-of-concept study, LA strain–volume characteristics provide incremental diagnostic value over conventional echocardiographic measures in the identification of elevated LV filling pressure.

## 1. Introduction

Evaluation of left ventricular filling pressure (LVFP) is integral to heart failure (HF) diagnosis and management. HF patients with elevated LVFP are prone to greater mortality and higher rates of hospitalization [1]. The hemodynamic definition of heart failure is based on either the presence of a reduced cardiac output or a normal cardiac output at the expense of elevated LVFP, either at rest or during exercise. Since some HF patients may show normal LVFP during rest but display a pathological rise during exercise, these patients constitute a particular diagnostic challenge.

In the setting of intermediate pretest likelihood but inconclusive diagnostic results in the resting state, right heart catheterization (RHC) with exercise or passive leg lift is recommended to establish HF diagnosis [2]. Passive leg lift (PLL) is a simple maneuver to aid in the ruling in or out of occult heart failure. PLL mimics a fluid challenge and results in an acute increase of approximately 300 mL of blood from the legs and mesenteric splanchnic pool to the central circulation [3]. Elevated pulmonary artery wedge pressure (PAWP) during PLL is thus indicative of HF despite normal PAWP at rest [4]. Therefore, when developing new non-invasive measures of LVFP, the ideal reference method should include the ability to detect patients with elevated LVFP both at rest and during provocation, i.e., either exercise or PLL.

In recent years, left atrial (LA) strain has evoked clinical interest as a robust, reproducible surrogate of LVFP in HF [5]. LA strain provides important information on LA function and was initially measured with tissue Doppler techniques but is now obtained from speckle tracking [6]. Hemodynamically, LA reservoir strain (LASr) is determined by LA relaxation, the descent of the mitral annulus, i.e., LV longitudinal systolic function, and LA compliance [7]. The diagnostic value of LASr in the detection of elevated LVFP has been recognized in several studies [8,9,10,11]. We have previously shown that LASr can effectively be incorporated into diagnostic algorithms for LVFP [12], and it is now recommended to be part of the standard assessment of LA function [13]. Given the strong dependence of LV systolic function, its use may be limited, for example, in patients with HF with preserved systolic function [6,7,14]. LA deformation during the cardiac cycle is modulated by intracardiac blood volume expansion and LA size, also recognized as a marker for diastolic dysfunction [15]. We hypothesized that the diagnostic value of LA strain could be further enhanced further by also taking the dynamic interplay between LA function and volume. Potentially, an HF patient with occult elevated LVFP could respond to volume challenge as in PLL either with reduced LA strain and/or with increased LA volume. Therefore, describing the relationship between simultaneously acquired LA strain and volume during PLL could provide incremental diagnostic information regarding LVFP.

In a proof-of-concept study, we explored the value of LA strain–volume loops and compared their diagnostic performance to detect elevated LVFP with conventional echocardiographic measures.

## 2. Methods

### 2.1. Patient Population

We retrospectively reviewed 39 adult patients with exertional dyspnea and clinical suspicion of HF who underwent simultaneous echocardiography and RHC at rest and during PLL. Patients with atrial fibrillation and non-diagnostic echocardiographic images were excluded. For comparison, we included 20 age-matched, healthy controls with no cardiovascular risk factors or medication. This study was approved by the local ethics committee, and all patients provided written informed consent.

### 2.2. Echocardiography

All echocardiographic examinations were performed by a single, experienced echocardiographer (PL) using commercial equipment (Vivid E9, GE Medical Systems, Horten, Norway) equipped with an adult 1.5–4.3 MHz phased array transducer. Echocardiography was performed simultaneously with RHC with the patient in a supine position. Standard views were obtained from the parasternal, apical, and subcostal windows in keeping with international recommendations [16]. Left ventricular (LV) volumes were measured using apical 4- and 2-chamber views, and ejection fraction was estimated using Simpson’s biplane method. LA volume was assessed in LA-focused apical views and indexed to body surface area (LAVI). LV stroke volume (SV) was assessed by multiplying LV outflow tract (LVOT) diameter during end-systole with a corresponding velocity–time integral obtained by placing a 5 mm Pulsed-wave Doppler (PW) sample volume in the LVOT in the apical 5-chamber view.

LV global longitudinal strain (LV GLS) and LA reservoir strain (LASr) were obtained by speckle tracking echocardiography in non-foreshortened apical views. LV GLS was obtained from three orthogonal apical views in keeping with current recommendations [17]. LASr was obtained in a non-foreshortened 4-chamber view. The LA endocardial border was traced, excluding the LA appendage and pulmonary veins. The region of interest (ROI) was visually inspected for tracking quality and repeated if found inadequate. Zero point was set at the onset of the QRS complex on the ECG. LASr was defined as the maximal inflection point above the baseline [18]. A non-invasive measure of LA compliance was calculated as LASr/E/e’ as proposed by earlier studies [19].

### 2.3. Right Heart Catheterization

RHC was performed by experts blinded to imaging results using a fluid-filled 6F Swan-Ganz catheter (Edwards Lifesciences, Irvine, CA, USA) employing jugular, medial cubital, or femoral access. Transducers were zeroed at mid-thoracic level in each patient. PAWP was measured at mid-A wave and averaged across five cardiac cycles. Elevated PAWP was defined as >15 mmHg, either at rest or at PLL. Stroke volume was measured by the thermodilution technique, and cardiac output was calculated. All pressure tracings were digitally stored, exported, and analyzed offline using standard hemodynamic software packages (WITT Series III, Witt Biomedical Corp., Melbourne, FL, USA).

### 2.4. LA Strain–Volume Loops Estimation

**Figure 1:**
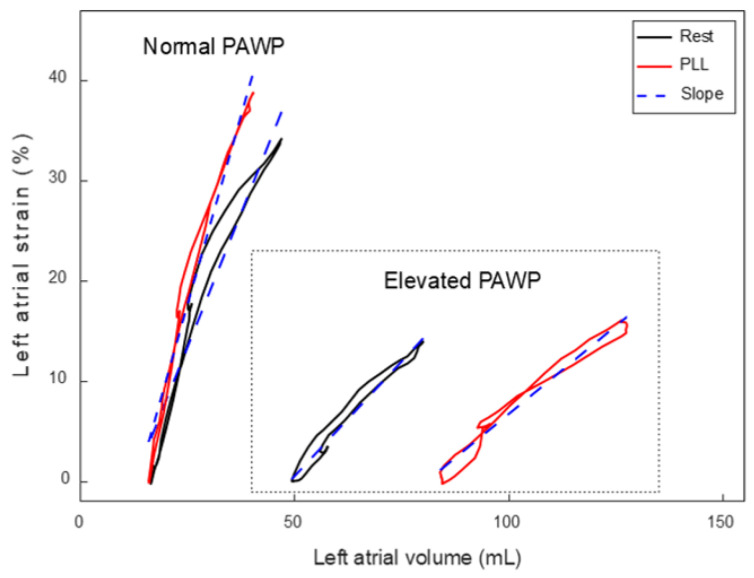
Left atrial (LA) strain-volume loops from simultaneous recording of LA strain and LA volumes obtained with speckle-tracking techniques in apical four-chamber view, at rest (blue) and passive leg lift (red). The loops displayed to the left are from a patient with normal left ventricular filling pressure (LVFP), and the two loops to the right from a patient with elevated LVFP. The dotted blue lines within each loop represent the LA strain-volume slope described by the best fit linear regression line. In the patient with normal LVFP, LA strain is increased during PLL but LA volumes similar, resulting in a higher slope at PLL than at rest. In the patient with elevated LVFP, a small increase in LA strain can be seen but minimal change in the slope.

Two-dimensional images of the LA were acquired from the apical 4-chamber view at rest and during PLL. Cine images were then transferred to TomTec (TomTec imaging GmbH, Unterschlessheim, Germany) for further strain analysis using LA strain software. Data from simultaneous strain and volume measurements were then exported and further analyzed using MATLAB (MathWorks Inc., Natick, MA, USA). LA strain volume loops were constructed by plotting lines between subsequent pairs of LA volume and LA strain samples from all included cardiac cycles in the exported data file. The slope of the LA strain volume loop was estimated through linear regression and expressed in %/mL. Figure 1 demonstrates examples of LA strain volume loops in one control with normal PAWP during both rest and at PLL and in a patient with normal PAWP at rest but elevated at PLL.

### 2.5. Statistics

Normality was tested using the Shapiro–Wilk test and visually reaffirmed using QQ plots. Continuous variables were expressed as median (interquartile range) and categorical variables as number and percentage. The Kruskal–Wallis test was used for the comparison of medians between multiple groups. When the null hypothesis was rejected (*p*-value < 0.05), a post-hoc analysis of inter-group comparisons was performed using the Mann–Whitney test. Using the Bonferroni correction for multiple testing, the null hypothesis was rejected if the *p*-value was <0.016. Correlations between invasive PAWP and echocardiographic variables were performed using Pearson’s r. Receiver operating characteristics (ROC) analysis was employed to assess the discriminative potential of the LA strain–volume relationship in the detection of elevated LVFP. This was described for the LA strain–volume slope with the area under the curve (AUC). Sensitivity, specificity, and accuracy ([true positives + true negatives]/total *n*) for the optimal threshold defined by the highest Youden index (sensitivity + specificity − 1) were described. IBM SPSS statistics version 23 and R version 4.3.1 (R Core Team) were used for statistical analysis. A *p*-value < 0.05 was considered statistically significant.

## 3. Results

The clinical and echocardiographic characteristics of the study population are presented in Table 1. Among HF patients, 58% (*n* = 18) had hypertension, 20% (*n* = 6) had diabetes mellitus, and 16% (*n* = 5) had ischemic heart disease. Thirty-five percent (*n* = 11) were on beta-blockers, fifty-one percent (*n* =16) were on ACE inhibitors, and 55% (*n* = 17) were on diuretics. Seven (22%) displayed LVEF ≤ 40%, 4 (13%) had an LVEF between 41 and 49%, and 20 (64%) had an LVEF ≥ 50%. HF patients displayed a higher proportion of men, were heavier, and had higher heart rates than controls. They displayed larger LV volumes, lower LVEF, and relatively poorer indices of diastolic dysfunction reflected in lower e’ velocity, higher E/e’ ratio, larger LA volumes, and lower LASr. LA strain–volume slopes at rest and PLL were lower in HF compared with controls.

Comparisons between subgroups based on elevation of PAWP with PLL are presented in Table 2. Among patients with HF, 15 (48%) displayed normal PAWP at rest and stress, 8 (26%) displayed normal PAWP at rest but elevated PAWP with PLL, and 8 (26%) displayed elevated PAWP during both rest and PLL. The comparison between groups revealed statistically significant differences in resting LV EF, LV GLS, LA volume, mitral valve deceleration time, LASr, and E/e’ with PLL (*p* < 0.05 for all comparisons). Resting E/e’ did not differ between groups. Both LA strain–volume slopes at rest and with PLL differed between groups. Intergroup comparisons revealed that the LA strain–volume slope at rest and during PLL was significantly lower in HF patients with elevated PAWP either at PLL or at rest compared to HF patients with normal PAWP (*p* = 0.005/0.004 and *p* = 0.002/0.002, respectively) (Table 2). LA strain at rest was significantly lower in patients with HF with elevated PAWP at rest compared to HF patients with normal PAWP (*p* = 0.005), but there was no significant difference between HF patients with normal PAWP and those with elevated PAWP only during PLL. No significant difference was observed in the intergroup comparisons for E/e’ at rest or during PLL.

### 3.1. Association of LA Strain–Volume Slope with Invasive PAWP

LA strain–volume slope displayed a moderately strong negative correlation with invasive pulmonary arterial wedge pressure (PAWP) both at rest and during PLL (r = −0.72 and −0.71, *p* < 0.001 for both) (Table 3). In comparison, LASr at rest and during PLL was also moderately correlated with PAWP, albeit with numerically less strong correlation (r = −0.64, *p* < 0.001, and r = −0.45, *p* = 0.01, respectively). E/e’, E/A, and mitral deceleration time displayed weaker correlations.

### 3.2. Diagnostic Performance of LA Strain–Volume Slope to Identify Elevated PAWP

**Figure 2:**
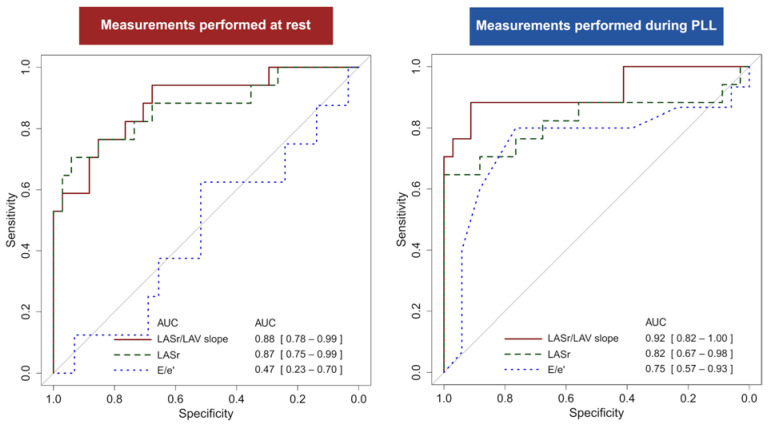
Diagnostic accuracy for LA strain–volume slope, LA strain, and E/e’ when acquired either at rest (**Left** panel) or during passive leg lift (PLL; **Right** panel) described by receiver operating curves and area under the curve (AUC) with 95% confidence intervals.

The diagnostic accuracy of the LA strain–volume slope at rest and during PLL is presented in Table 4 and in Figure 2. At a 0.74%/mL cut-off, the LA strain–volume slope displayed 88% sensitivity and 91% specificity to identify elevated PAWP with PLL (AUC 0.92 [0.82–1.00]). A numerically worse diagnostic accuracy was observed for the LA strain–volume slope acquired at rest. In comparison, LASr alone demonstrated a strong but numerically worse diagnostic performance (at rest: AUC 0.87 [0.75–0.99]; PLL: AUC 0.82 [0.67–0.98]). Mitral E/e’, E/A, and mitral deceleration time showed poorer performance.

## 4. Discussion

In this proof-of-concept study, the LA strain–volume relationship displayed a significant association with PAWP measured by catheterization during PLL and superior diagnostic performance to distinguish normal from elevated filling pressure when compared with conventional echocardiographic surrogates such as mitral E/e’ in addition to deformation measures such as LV GLS and LASr. To our knowledge, this is the first study to explore the potential utility of simultaneously acquired LA strain–volume information to assess LV filling pressure in HF patients. While our findings need to be validated in larger populations, they suggest an important and potential role for the LA strain–volume relationship as a novel, non-invasive distinguisher to disclose both overt and occult LVFP elevations. In contrast to other echocardiographic indices of elevated PAWP, the LA strain–volume slope was significantly lower in patients with elevated PAWP compared to patients with HF patients with normal PAWP. This further suggests a potentially incremental role of the LA strain–volume slope to conventional echocardiographic measurements. The LA strain–volume slope during PLL provided the highest diagnostic accuracy; however, the small sample size limits definitive conclusions.

PLL has been proposed as a simple, preload-enhancing provocative measure to unmask occult elevated filling pressures in earlier studies [20,21]. In an RHC study including patients with dyspnea and suspected HF, PLL displayed a 91% sensitivity and 92% specificity in identifying exercise-exacerbated PAWP [21]. While echocardiography is valuable in routine clinical practice to raise suspicion of occult disease, current recommendations suggest limited use of echocardiographic measures in the absence of significantly depressed LV function [22]. A majority of patients in our HF cohort displayed preserved EF, which may explain why conventional indices of filling pressure, such as resting mitral E/e’, did not differ between groups. Despite this presentation, the LA strain–volume relationship distinguished these groups, suggesting promise as a non-invasive distinguisher in the setting of preserved EF. This needs to be validated in larger studies.

In agreement with the HFA-PEFF algorithm, ref. [23] however, LV GLS, LAVI, and mitral E/e’ during preload stress displayed greater promise to distinguish patients with a disproportionate rise in filling pressures when provoked.

Recent studies suggest that novel indices obtained by echocardiography considering the relationship between LV [24] or LA inflow and outflow velocities [25] provide accurate and complimentary information on LV filling status in the setting of preserved EF. Our findings introduce the dynamic interplay between LA pressure and volume as a novel surrogate of LV filling pressure during provocation. However, given the small sample size that deterred further sub-analysis owing to limited statistical power, these findings are hypothesis-generating at best and provide direction for further studies validating these findings in larger cohorts.

LASr has been proposed as a useful complement to the assessment of LV filling pressures in recent times [12]. However, the relation of LASr with LA pressure is modulated by other hemodynamic factors and limits its prediction of LV filling pressures in patients with normal EF. First, the association between LASr and LV filling pressures is indirect as it is mediated by the association of LA chamber stiffness with LA V-wave pressure [7]. Second, LV long axis function is a strong determinant of LASr, making the assessment of diastolic status challenging when LV GLS is preserved [26]. While the interplay between LA volume and deformation offers value as a promising adjunct to LV filling pressure assessment, further studies are needed to address the limitations of LASr as a stand-alone measure. Despite the small sample size, we believe that the results of our study display promising clinical utility and warrant further exploration. Noninvasive estimators of elevated filling pressure during provocation have strong clinical utility for HFpEF diagnosis, regulation of medical therapy, stratification to reference-standard catheterization, and outcome prediction.

Certain limitations must be acknowledged. This was a retrospective, single-center, observational study on a small number of patients. The small sample size limits the ability to conclude whether LA strain–volume slopes at rest or during PLL outperforms LASr and emphasizes the need for replication of our findings in larger studies, as well as studies in different HF subtypes, e.g., HF with reduced and preserved ejection fraction. Thus, this study can only be considered a proof-of-concept study. Nonetheless, even with the small sample size, intergroup comparisons resulted in statistically significant differences in LA strain–volume slope between HF patients with different PAWP status. All HF patients were on medications which could potentially alter hemodynamic status. The low prevalence of HF patients with reduced EF limits the extension of our findings to a general HF population. The heterogeneity of the HF population further limits the interpretation of our results. Although subjects in the control group were presumed to have normal PAWP, these healthy volunteers did not undergo RHC, adding some uncertainty to the results. The healthy volunteers were added to this proof-of-concept study to improve power based on a presumed high likelihood that they had normal PAWP both at rest and during PLL. Importantly, the LA strain–volume slope did provide the most significant intergroup differences among HF patients with different PAWP status, i.e., among patients who all underwent RHC

## 5. Conclusions

In this proof-of-concept study, LA strain–volume characteristics provide incremental diagnostic value over conventional echocardiographic measures in the identification of elevated LV filling pressure during PLL.

## Figures and Tables

**Table 1:** Clinical characteristics and echocardiographic data for study population. Data presented as median [Q1; Q3] or number (%).

	Controls (*n* = 20)	Heart Failure (*n* = 31)
Age, yrs	59 [51, 65]	67 [55, 75]
Female, %	13 (65)	8 (26)
Heart rate, min^−1^	66 [61, 72]	70 [56, 81]
Height, cm	173 [165, 181]	168 [162, 174]
Weight, kg	72 [64, 81]	82 [71, 88]
Systolic blood pressure, mmHg	130 [130, 130]	130 [121, 148.50]
Diastolic blood pressure, mmHg	85 [85, 85]	78 [73, 81]
Comorbidities, *n* [%]		
**Hypertension**		18 [58]
Diabetes		6 [20]
Ischemic heart disease		5 [16]
Laboratory findings		
NT pro-BNP, ug/L		593 [163, 1136]
**Medications, *n* [%]**		
Beta-blocker		11 [35]
ACEi/ARB		16 [51]
Diuretics		17 [55]
**Echocardiography**		
LVIDd, mm	47 [44, 49]	50 [47, 54]
LVIDs, mm	31 [28, 33]	34 [32, 38]
IVSd, mm	10 [9, 10]	10 [9, 12]
PWDd, mm	7 [7, 8]	8 [7, 10]
Relative wall thickness	0.32 [0.29, 0.38]	0.33 [0.3, 0.36]
LVEDV, mL	83 [73, 105]	86 [65, 112]
LV EF, %	60 [56, 64]	50 [45, 61]
LV GLS at rest, %	−19 [−20, −18]	−18 [−21, −15]
LV GLS at PLL, %	−19 [−20, −17]	−19 [−21, −15]
LA volume, mL	45 [34, 50]	68 [45, 80]
Stroke volume, mL	74 [60, 84]	61 [40, 74]
Mitral E/A ratio at rest	1.0 [0.86, 1.34]	1.1 [0.83, 1.40]
Mitral deceleration time	157 [128, 196]	164 [108, 193]
Mitral e’ avg, cm/s	7.7 [6.5, 10.1]	6 [6, 7]
Mitral E/e’ ratio at rest	8 [6, 10]	11 [9, 12]
Mitral E/e’ ratio at PLL	7 [6, 8]	11 [7, 13]
LASr at rest, %	41 [32, 47]	25 [17, 37]
LASr at PLL, %	37 [33, 44]	25 [15, 36]
Slope at rest, %/mL	1.26 [1.01, 1.50]	0.81 [0.52, 1.07]
Slope at PLL, %/mL	1.22 [1.02, 1.50]	0.71 [0.48, 1.00]
**Right heart catheterization**		
PAWP_rhc_ at rest, mmHg	-	12 [8, 13.50]
PAWP_rhc_ with PLL, mmHg	-	17 [12, 21]
Cardiac output_rhc_ att PLL, L/min	-	5.4 [3.8, 6.2]
Cardiac output_rhc_ at rest, L/min	-	4.90 [3.88, 5.53]

BNP, B-type natriuretic peptide; LVID, left ventricular internal diameter; d, diastole; s, systole; IVS, interventricular septum; PWD, posterior wall dimension; LVEDV, left ventricular end-diastolic volume; LV, left ventricle; EF, ejection fraction; GLS, global longitudinal strain; LA left atrium; LASr, left atrial reservoir strain PLL, passive leg lift; PAWP, pulmonary artery wedge pressure.

**Table 2:** Clinical characteristics, echocardiographic, and right heart catheterization data for the study population, stratified based on elevation of PCWP with PLL. Healthy controls were assumed to have a normal PAWP at rest and with PLL. Data presented as median (Q1, Q3) or number (%).

	Healthy Controls (*n* = 20)	Heart Failure Patients						
		Normal PAWP at Rest and PLL Group 1 (*n* = 15)	Normal PAWP at Rest/Elevated at PLL Group 2 (*n* = 8)	Elevated PAWP at Rest Group 3 (*n* = 8)	Overall	Group 1 vs. 2	Group 1 vs. 3	Group 2 vs. 3
Age, yrs	59 [52–65]	67 [52–75]	65 [56–77]	69 [56–76]	0.43			
Male, %	13 (65.0)	5 (33.3)	2 (25.0)	1 (12.5)	0.034	0.99	0.56	0.99
Heart rate, bpm	66 [61–72]	65 [59–80]	73 [59–81]	74 [72–84]	0.43			
Height, cm	174 [165–181]	172 [163–175]	167 [161–170]	169 [161–174]	0.26			
Weight, kg	72 [64–81]	83 [73–88]	74 [70–84]	86 [78–89]	0.14			
Systolic BP, mmHg	-	130 [127–140]	130 [122–148]	139 [117–158]	0.99			
Diastolic BP, mmHg	-	80 [73–88]	76 [72–79]	78 [74–81]	0.42			
NT pro-BNP, ug/L	-	461 [153–886]	694 [316–1491]	744 [147–1412]	0.52			
**Echocardiography**								
LVIDd, mm	47 [44–49]	48 [44–52]	52 [50–56]	50 [50–54]	0.032	0.12	0.37	0.60
LVIDs, mm	31 [28–33]	36 [33–40]	28 [28–33]	33 [33–36]	0.11			
IVSd, mm	10 [9–10]	10 [9–11]	10 [9–12]	12 [10–14]	0.39			
PWDd, mm	8 [7–8]	8 [7–8]	10 [8–11]	9 [8–9]	0.028	0.19	0.10	0.56
LVEDV, mL	83 [73–105]	83 [66–90]	105 [76–111]	111 [62–120]	0.91			
LV EF, %	60 [56–64]	60 [50–65]	45 [40–48]	45 [39–55]	0.001	0.004 *	0.025	0.87
LV GLS, rest, %	−19 [−20–−18]	−20 [−23–−18]	−18 [−18–−16]	−12 [−18–−10]	0.039	0.20	0.028	0.20
LV GLS, PLL, %	−19 [−20–−18]	−19 [−21–−17]	−19 [−20–−15]	−12 [−22–−11]	0.74			
LA volume, mL	45 [34–50]	50 [37–69]	71 [55–82]	79 [69–105]	<0.001	0.093	0.012 *	0.32
Stroke volume, mL	74 [60–84]	61 [39–73]	61 [44–73]	56 [42–77]	0.29			
Mitral E/A	1.0 [0.9–1.3]	0.9 [0.8–1.2]	1.0 [0.9–1.2]	1.7 [1.1–2.5]	0.71			
Mitral dec time, ms	158 [129–196]	183 [133–221]	164 [118–179]	105 [94–112]	0.025	0.43	0.011 *	0.026
Mitral e’ avg, cm/s	8 [7–10]	7 [6–7]	6 [5–7]	6 [6–7]	0.046	0.36	0.73	0.55
Mitral E/e’ rest	8 [6–10]	11 [11–14]	8 [7–10]	10 [8–12]	0.056	0.17	0.54	0.77
Mitral E/e’ PLL	7 [6–8]	8 [6–7]	13 [11–16]	12 [9–13]	0.008	0.045	0.27	0.31
LASr, rest, %	41 [32–47]	35 [29–38]	17 [10–27]	17 [12–20]	<0.001	0.020	0.005 *	0.67
LASr, PLL, %	37 [33–44]	34 [29–38]	12 [9–22]	17 [10–25]	<0.001	0.004 *	0.020	0.75
Slope rest, %/mL	1.26 [1.13–1.45]	1.07 [0.83–1.34]	0.87 [0.77–1.02]	0.57 [0.37–0.88]	<0.001	0.005 *	0.002 *	0.53
Slope PLL, %/mL	1.22 [1.02–1.50]	0.93 [0.77–1.13]	0.71 [0.39–1.15]	0.35 [0.24–0.87]	<0.001	0.004 *	0.002 *	0.99
**Right heart catheterization**								
PAWP rest, mmHg	-	8 [7–11]	12 [11–13]	18 [13–19]	0.001	0.018	0.001 *	0.045
PAWP PLL, mmHg	-	12 [10–13]	19 [18–20]	23 [22–25]	<0.001	<0.001 *	<0.001	0.015 *
CO rest, L/min	-	5.0 [4.4–5.5]	5.0 [4.3–5.5]	3.6 [3.3–5.4]	0.21			
CO PLL, L/min	-	5.4 [4.6–5.9]	5.4 [4.2–6.2]	4.6 [3.6–6.0]	0.85			

BNP: B-type natriuretic peptide; BP: blood pressure; dec: deceleration; CO: cardiac output; LVID: left ventricular internal diameter: diastole; s: systole; IVS: interventricular septum; PWD: posterior wall dimension; LVEDV: left ventricular end-diastolic; LV: left ventricle; EF: ejection fraction; GLS: global longitudinal strain; LA: left atrium; LASr: left atrial reservoir strain; PLL: passive leg lift; PAWP: pulmonary artery wedge pressure.* denotes significance after Bonferroni correction (*p* < 0.016).

**Table 3:** Correlation between different echocardiographic indices of elevated left ventricular filling pressures and invasively measured pulmonary artery wedge pressure during passive leg lift.

	r	*p*
LA strain–volume slope, rest	−0.72	<0.001
LA strain–volume slope, PLL	−0.71	<0.001
LA strain, rest	−0.64	<0.001
LA strain, PLL	−0.45	0.011
E/e’ rest	−0.11	0.61
E/e’ PLL	0.27	0.15
E/A	0.33	0.20
Mitral deceleration time	−0.43	0.022

Abbreviations: LA: left atrial; PLL: passive leg lift.

**Table 4:** Diagnostic accuracy for different echocardiographic indices in detection of elevated invasively measured pulmonary artery wedge pressure during passive leg lift.

	AUC [95% CI]	Optimal Threshold	Accuracy (%)	Sensitivity (%)	Specificity (%)
LA strain–volume slope, rest, %/mL	0.89 [0.75–1.00]	<1.07	76	94	68
LA strain–volume slope, PLL, %/mL	0.92 [0.82–1.00]	<0.74	90	88	91
LA strain, rest, %	0.87 [0.75–0.99]	<22	86	71	94
LA strain, PLL, %	0.82 [0.67–0.98]	<20	88	65	100
E/e’ rest	0.47 [0.23−0.70]	>10	55	63	52
E/e’ PLL	0.75 [0.57–0.93]	>8	78	80	76
E/A	0.62 [0.38–0.86]	>0.9	49	88	38
Mitral deceleration time, ms	0.66 [0.46–0.85]	<115	77	50	88

Abbreviations: AUC: area under the curve; CI: confidence interval; LA: left atrial; PLL: passive leg lift.

## Data Availability

Data is available under reasonable request from the corresponding author.
